# Unconventional multidisciplinary team strategy for tracheostomy in COVID‐19

**DOI:** 10.1002/anr3.12074

**Published:** 2020-11-17

**Authors:** T. G. Smith, I. Ahmad, A. Takhar, P. Surda, K. El‐Boghdadly

**Affiliations:** ^1^ Department of Anaesthesia Guy's and St Thomas' NHS Foundation Trust London UK; ^2^ Centre for Human and Applied Physiological Sciences King's College London London UK; ^3^ King's College London London UK; ^4^ Department of Otorhinolaryngology Guy's and St Thomas' NHS Foundation Trust London UK; ^5^ Department of Otorhinolaryngology Guy's and St Thomas' NHS Foundation Trust London UK

**Keywords:** aerosolisation, airway management, COVID‐19, multidisciplinary team, percutaneous tracheostomy

The approach an institution takes to tracheostomy during the coronavirus disease 2019 (COVID‐19) pandemic has broad potential to influence morbidity and mortality, as tracheostomy is at the intersection of patient‐centred care, healthcare worker safety and resource allocation [1]. Consensus guidance is, by necessity, based largely on expert opinion, and real‐world experience is required [1]. We have accumulated extensive experience using an unconventional multidisciplinary team strategy in which percutaneous dilatational tracheostomy is undertaken at the bed‐side in open intensive care units (ICUs) by teams combining anaesthetists and surgeons. In the context of a strained critical care system, greater resource efficiency is a crucial benefit of this approach.

Our institution has been at the epicentre of the UK pandemic, admitting hundreds of patients to regular and ‘surge’ ICUs. There has been a very high requirement for tracheal intubation and mechanical ventilation, and subsequently for tracheostomy. We established mobile emergency rapid intubation teams (MERIT) early in the pandemic response, which follow specific protocols for personal protective equipment (PPE) and minimising aerosol generation and thus operator risk of infection [2]. Mobile emergency rapid intubation teams, consisting of two consultant anaesthetists and two assistants, are responsible for all COVID‐19 tracheal intubations in the hospital [3]. Our key multidisciplinary innovation was to then develop a dedicated tracheostomy team comprising two ear, nose and throat (ENT) surgeons and two MERIT anaesthetists. To our knowledge, we were the first UK centre to develop such teams and publish guidelines for tracheostomy in COVID‐19 including procedures for PPE, minimising aerosolisation and improved success with minimal complications [4].

ENT surgeons have been involved in ‘surge’ tracheostomy strategies in other centres, for example, instituting a dedicated tracheostomy theatre. However, our approach was distinct and unusual both in combining ENT surgeons with anaesthetists to form a tracheostomy team and, crucially, in this team undertaking percutaneous tracheostomies independently at the bed‐side in the ICU. This was in contrast to normal practice where tracheostomies would be inserted either by ICU operators alone, or alternatively in the operating theatre as a surgical tracheostomy.

The ENT/MERIT tracheostomy team has quickly developed expertise in using advanced PPE and minimising aerosolisation in accordance with carefully devised procedures including a step‐by‐step protocol for bed‐side tracheostomy (Fig. 1). This protocol includes the use of ultrasound to identify structures and mark a ‘never above’ line to minimise the risk of damaging the tracheal tube cuff, as well as videolaryngoscopy to view the tracheal tube cuff and fibreoptic bronchoscopy for internal viewing. Our self‐contained team model promotes efficient use of personnel and resources by relieving ICU staff and avoiding ICU‐theatre transfers. At our institution, it has also exploited changing capacity of MERIT anaesthetists as the requirement for tracheostomies has progressively overtaken primary intubations. Outcomes from our first 28 tracheostomies have been favourable with no significant complications or deaths, and no operators have developed COVID‐19 (GSTT service evaluation ID 10780; Table 1).

**Figure 1:**
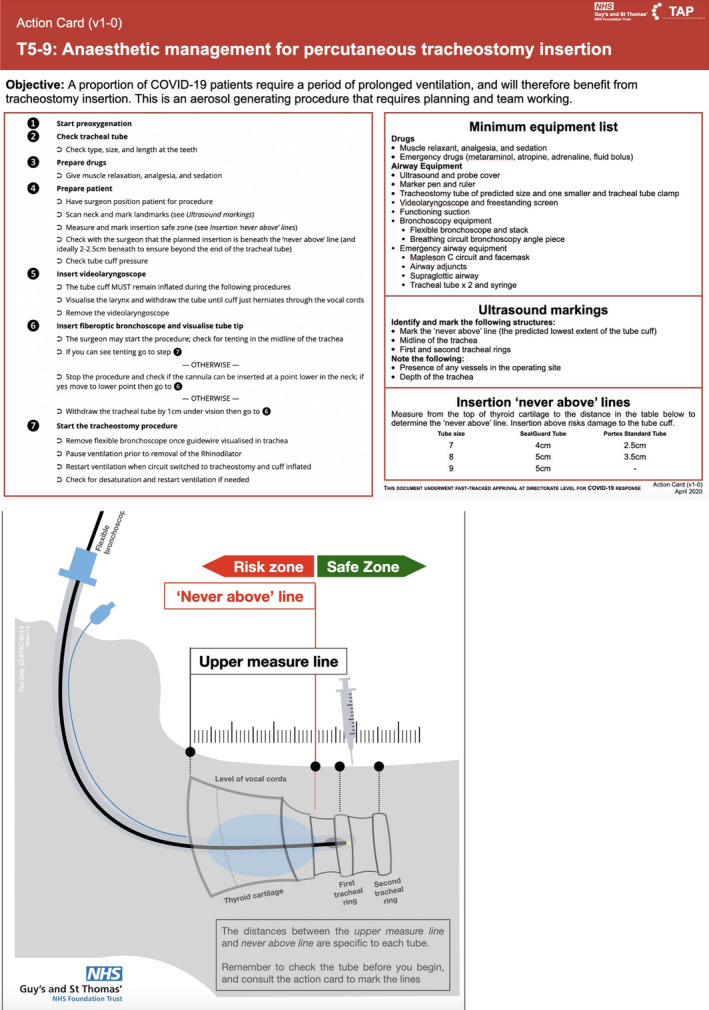
Anaesthetic management for percutaneous tracheostomy insertion in COVID‐19 at Guy's and St Thomas' NHS Foundation Trust. (a) Trust's action card including step‐by‐step protocol, minimum equipment list and instructions for ultrasound markings. (b) Schematic for measuring structures using ultrasound and marking a ‘never above’ line to minimise the risk of damaging the tracheal tube cuff.

**Table 1:** Data from 28 patients with COVID‐19 undergoing percutaneous tracheostomy in ICU by dedicated multidisciplinary teams.

Variable	Median (IQR [range]) or n (%)
Age (years)	55 (48–61 [28–78])
Sex	
Male	18 (64%)
Female	10 (36%)
Ethnicity	
Black, Asian or minority ethnic group	20 (71%)
White	8 (29%)
Weight (kg)	76 (72–95 [55–118])
Height (m)	1.75 (1.65–1.79 [1.52–1.93])
Body mass index (kg.m^−2^)	29 (24–31 [22–40])
APACHE II score on admission	13 (12–16 [8–19])
Timing of tracheostomy (days post‐intubation)	19 (14–24 [6–31])
Respiratory status at time of tracheostomy	
PEEP (cmH_2_O)	8 (6–8 [5–10])
FiO_2_ (%)	0.35 (0.30–0.40 [0.21–0.55])
PaO_2_ (mmHg)	72 (68–79 [59–102])
P/F ratio	207 (183–246 [144–379])
Duration of follow‐up (days post‐tracheostomy)	40 (45–49 [9–70])
Patient status at follow‐up	
Alive	28 (100%)
Discharged from ICU	24 (86%)
Decannulated	24 (86%)
Discharged from hospital	18 (64%)
Days from tracheostomy to	
Discharge from ICU	20 (13–28 [4–48])
Decannulation	18 (14–30 [9–46])
Discharge from hospital	37 (28–45 [13–63])
Significant complications from tracheostomy^*^	0 (0%)
Transmission of COVID‐19 to operators	0 (0%)
Mortality	0 (0%)

^*^Significant complications are defined as Clavien‐Dindo Grade 2 or higher.

APACHE II, acute physiology and chronic health evaluation II score; FiO_2_, fraction of inspired oxygen; PaO_2_, arterial partial pressure of oxygen; PEEP, positive end‐expiratory pressure; P/F ratio, PaO_2_/FiO_2_ ratio.

The presence of ENT surgeons in the team may have allowed more challenging tracheostomies that would not normally be attempted in ICU (e.g. obese patients) to still be undertaken in the most resource‐efficient manner. While the ideal location for performing a tracheostomy on a COVID‐19‐positive patient is in a negative pressure side room or operating theatre, this may not be available, and transferring critically ill patients to theatre also has major resource and safety implications. Recent preliminary data from a national UK audit (UK COVIDTrach) indicate that percutaneous tracheostomy is at least as safe as surgical tracheostomy in COVID‐19 [5]. Anecdotally, percutaneous tracheostomies may be less prone to wound site infections and dislodgement in the presence of thick tenacious secretions in COVID‐19.

Until further data are forthcoming, we recommend consideration of this multidisciplinary team model and protocol for tracheostomy in COVID‐19 in order to deliver safe patient‐centred care and support healthcare worker safety while simultaneously optimising the balance between these priorities and the efficient utilisation of scarce resources.

## References

[1] McGrath BA , Brenner MJ , Warrillow SJ , et al. Tracheostomy in the COVID‐19 era: global and multidisciplinary guidance. Lancet Respiratory Medicine 2020; 8: 717–25. 32422180 10.1016/S2213-2600(20)30230-7 PMC7228735

[2] Ahmad I , Wade S , Langdon A , Chamarette H , Walsh M , Surda P . Awake tracheal intubation in a suspected COVID‐19 patient with critical airway obstruction. Anaesthesia Reports 2020; 8: 28–31 32373789 10.1002/anr3.12041 PMC7197305

[3] Ahmad I , Jeyarajah J , Nair G , et al. A prospective, observational, cohort study of airway management of patients with COVID-19 by specialist tracheal intubation teams. Canadian Journal of Anesthesia 2020; ePub 4 September. doi: 10.1007/s12630-020-01804-3. PMC7472940 32886298

[4] Takhar A , Walker A , Tricklebank S , et al. Recommendation of a practical guideline for safe tracheostomy during the COVID‐19 pandemic. European Archives of Otorhinolaryngology 2020; 277: 2173–84. 32314050 10.1007/s00405-020-05993-x PMC7170707

[5] Hamilton NJI , Jacob T , Schilder AGM , et al. COVIDTrach; the outcomes of mechanically ventilated COVID‐19 patients undergoing tracheostomy in the UK: Interim Report 22nd May 2020. https://www.entuk.org/sites/default/files/COVIDTrach%20-%20Interim%20Report%2C%2022%20May%202020.pdf (accessed 02/06/2020). 10.1002/bjs.12020 PMC7929177 32940347

